# Assessment of microsatellite and SNP markers for parentage assignment in ex situ African Penguin (*Spheniscus demersus*) populations

**DOI:** 10.1002/ece3.1600

**Published:** 2015-09-19

**Authors:** Christiaan Labuschagne, Lisa Nupen, Antoinette Kotzé, Paul J. Grobler, Desiré L. Dalton

**Affiliations:** ^1^ Department of GeneticsUniversity of the Free StateP.O. Box 339Bloemfontein9300South Africa; ^2^ Inqaba Biotechnical Industries (Pty) LtdP.O. Box 14356Hatfield0028South Africa; ^3^ National Zoological Gardens of South AfricaP.O. Box 754Pretoria0001South Africa; ^4^ Department of Biological SciencesPercy FitzPatrick InstituteUniversity of Cape TownRondeboschCape Town7701South Africa

**Keywords:** African penguin, ex situ populations, exclusion‐based paternity, pedigree

## Abstract

Captive management of ex situ populations of endangered species is traditionally based on pedigree information derived from studbook data. However, molecular methods could provide a powerful set of complementary tools to verify studbook records and also contribute to improving the understanding of the genetic status of captive populations. Here, we compare the utility of single nucleotide polymorphisms (SNPs) and microsatellites (MS) and two analytical methods for assigning parentage in ten families of captive African penguins held in South African facilities. We found that SNPs performed better than microsatellites under both analytical frameworks, but a combination of all markers was most informative. A subset of combined SNP (*n* = 14) and MS loci (*n* = 10) provided robust assessments of parentage. Captive or supportive breeding programs will play an important role in future African penguin conservation efforts as a source of individuals for reintroduction. Cooperation among these captive facilities is essential to facilitate this process and improve management. This study provided us with a useful set of SNP and MS markers for parentage and relatedness testing among these captive populations. Further assessment of the utility of these markers over multiple (>3) generations and the incorporation of a larger variety of relationships among individuals (e.g., half‐siblings or cousins) is strongly suggested.

## Introduction

The growing role of captive institutions in the conservation of threatened species requires that they maintain sustainable and genetically diverse ex situ populations that can meaningfully contribute to in situ conservation (Lacy et al. 2013). Molecular tools have the potential to complement and validate traditional studbook‐based genetic management of captive populations, with the goal of reducing the negative effects of inbreeding and loss of genetic diversity (Putnam and Ivy 2013). Complete pedigrees are required to effectively manage the genetic status of captive populations (Ivy and Lacy 2010), but these are not always available, as the parentage of offspring is often uncertain (Putnam and Ivy 2013).

The endangered African penguin (*Spheniscus demersus*) is endemic to southern Africa, with 25 breeding colonies distributed along the coastline between central Namibia and St. Croix Island (Algoa Bay, South Africa). The population is declining despite multiple conservation interventions (IUCN Red List, BirdLife International, 2013) with an estimated 26,000 breeding pairs left (Crawford et al. 2011). Declines have been attributed to excessive egg and guano harvesting (Shelton et al. 1984), competition for food with seals (Crawford et al. 1992) and commercial fisheries (Frost et al. 1976), oil spills (Morant et al. 1981; Adams 1994; Underhill et al. 1999), loss of habitat, and climate change affecting prey distribution (Boersma 2008; Crawford et al. 2011).

African penguins breed well in captivity and are currently held in 11 zoos and aquariums across South Africa. Ex situ populations serve a number of different roles in conservation efforts including public education, resources for scientific discovery, and sources for supplementation or restoration of in situ populations (Lacy 2009). The latter has recently been identified as a potentially valuable conservation action, and looks likely to be implemented in the near future, necessitating a sound understanding of the genetic status of the captive populations. The African Association of Zoos and Aquaria (PAAZA) established a regional studbook as part of their African Preservation Programme as part of the ex situ management of this species. Similar to other studbooks, it uses the Single Population Analysis and Record Keeping System (SPARKS) developed by the International Species Information System (ISIS) and the PM2000 database program (Pollack et al.*,* 2002). Studbook‐based analyses indicated that 70.9% of the full pedigree information is known and that the population mean kinship is 0.02 (African Penguin Regional Studbook, 2011). The use of molecular methods to confirm parentage and analyze relatedness among ex situ individuals will complement studbook‐based genetic management of the African penguin captive population.

Genealogical relationships among individuals in a population represent a simple concept in biology, but can be powerful when applied to answer evolutionary and ecological questions (Hauser et al. 2011). Pedigree information plays a central role in the study of diverse ecological and evolutionary topics, such as sexual selection, patterns of dispersal and recruitment, quantitative genetic variation, mating systems, and managing the conservation of populations of endangered species (Wang and Santure 2009; Jones et al. 2010). Molecular markers provide new possibilities in establishing genealogical relationships among individuals in populations where such information is difficult to collect from field observations (Pemberton 2008).

Microsatellites (MS) have been the marker of choice for parental assignment and reconstruction, owing to their high polymorphic information content (PIC) and wide availability (Glowatzki‐Mullins et al. 1995; Hauser et al. 2011). However, these markers have several disadvantages including homoplasy, complex mutational patterns, and data analysis may be affected by genotyping errors (Angers et al., 2000; Hoffman et al., 2005). Despite being bi‐allelic, resulting in lower resolving power, single nucleotide polymorphisms (SNPs) are becoming increasingly popular (Baruch and Weller 2008; Hauser et al. 2011) due to their low genotyping error rate (<0.1%), high‐throughput screening applications, and the fact that SNPs are easier and cheaper to standardize between laboratories compared to microsatellites (Anderson and Garza 2006).

In parallel to the advances in genetic markers, many statistical methods have been proposed to analyze marker data for pedigree information (Jones and Ardren 2003). Jones et al. (2010) categorized parentage analysis techniques into six categories, namely exclusion, categorical allocation, fractional allocation, full probability parentage analysis, parental reconstruction, and sibship reconstruction. Exclusion‐based methods compare the compatibility of offspring and parental genotypes with Mendelian inheritance, so that a putative parent is rejected as a true parent if both alleles at one locus mismatch with that of an offspring (Jones et al. 2010). Exclusion methods are appealing as they are simple in concept and implementation and quick in computation and do not require allele frequency information (Wang 2012). However, exclusion methods suffer from several weaknesses including false exclusion due to genotyping errors, valuable marker information is not fully utilized and exclusion rules are necessary, but insufficient for relationship inference (Jones et al. 2010; Wang 2012). A range of likelihood methods have been developed that seek to overcome these problems by determining probabilities of parentage assignment from simulations, Monte Carlo permutations or Bayesian approaches (Jones et al. 2010). Likelihood‐based methods employ Mendel's laws quantitatively to calculate the likelihoods of different candidate relationships among a set of individuals and choose the relationship that has the highest likelihood as the best inference (Wang 2012).

In this study, we compare the power of parentage assignment of 31 SNPs and 12 MS markers in isolation and in combination in captive populations of African penguins. Development of a marker set that accurately determines parentage will provide information on the relationships and relatedness among individuals (e.g., extra‐pair mating) and contribute to the management of captive African penguins worldwide.

## Materials and Methods

### Pedigrees and sampling

**Figure 1:**
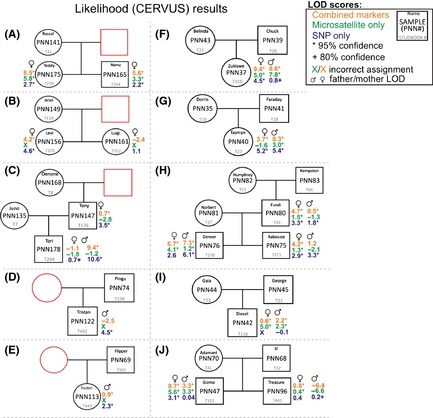
Studbook‐based pedigrees of ten families of African penguins (*Spheniscus demersus*) based on data from Single Population Analysis and Record Keeping System (SPARKS) superimposed with parentage assignment data from CERVUS (likelihood). Squares indicate males, circles indicate females, and red shapes indicate unsampled individuals.

**Figure 2:**
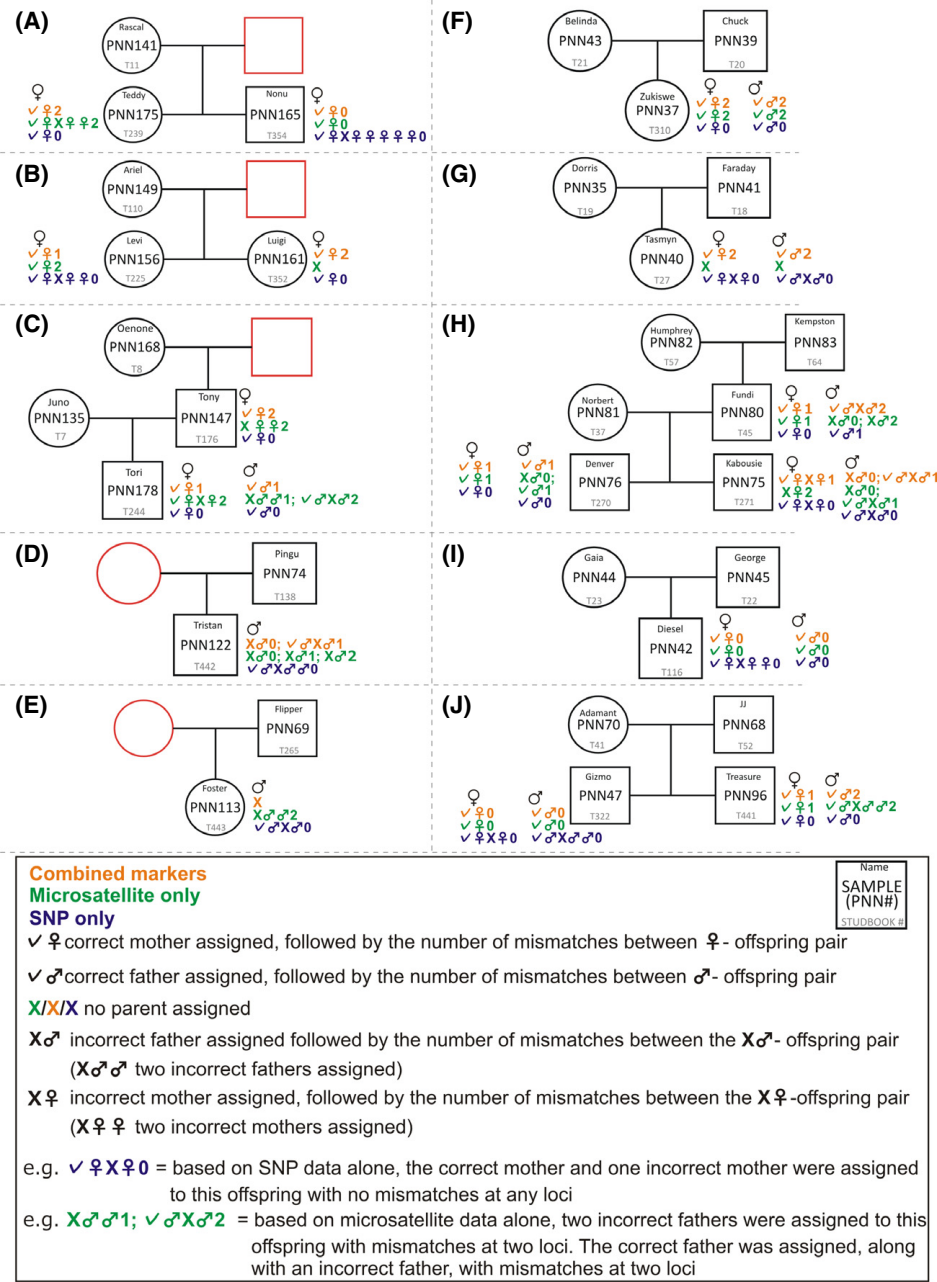
Studbook‐based pedigrees of ten families of African penguins (*Spheniscus demersus*) based on data from SPARKS superimposed with parentage assignment data from PARFEX (exclusion). Squares indicate males, circles indicate females, and red shapes represent unsampled individuals.

Blood samples were collected from 33 African penguins, which are housed in three captive facilities in South Africa: the Two Oceans Aquarium (Cape Town), the National Zoological Gardens of South Africa (Pretoria), and uShaka Sea World (Durban). All penguins are part of the permanent breeding population. Ten family‐group pedigrees were constructed based on the regional studbook data (SPARKS) as shown in Figures 1 and 2 (A–J).

### Molecular gender verification

For each individual, 30 *μ*L of blood was collected on FTA paper. DNA was extracted using the Qiagen DNeasy^®^ (Qiagen, Valencia, CA) Blood and Tissue Kit. The extraction protocol as outlined in the manufacturer's protocol was followed. Chromo Helicase DNA CHD (chromo‐helicase‐DNA‐binding) gene‐binding gene‐based molecular sexing was conducted using the 2550F/2718R (Fridolfsson and Ellegren 1999) primer set. Promega GoTaq^®^ Flexi DNA polymerase (Promega Corporation) Promega, Madison, WI was used for amplification in 25 *μ*L reactions. The final reaction conditions were as follows: 1× PCR buffer, 1.5 mmol/L MgCl_2_, 200 *μ*mol/L of each dNTP, 5 pmol of each of the forward and reverse primer, 0.25 U Taq DNA polymerase, and 10–20 ng genomic DNA template. A no template control as well as positive controls for a male and female bird of known sex was included. The conditions for PCR amplification were as follows: initial denaturation for 2 min (min) at 95°C, 30 cycles for 30 sec (sec) at 95°C, 30 sec at 50°C, and 2 min at 72°C, followed by final extension at 72°C for 10 min. The PCR was carried out in the BOECO TC‐PRO Thermal Cycler. Amplicons were separated by electrophoresis in a 2% agarose gel for 45 min at 100 V in 1× Tris‐borate‐EDTA buffer. A single‐band pattern was considered male (CHD‐Z), while the two‐band pattern was considered female (CHD‐W/CHD‐Z).

### Microsatellite genotyping

A total of 12 microsatellite markers were typed as described in Schlosser et al. (2003) and Labuschagne et al. (2013). Promega GoTaq^®^ Flexi DNA polymerase (Promega Corporation) was used for amplification in 12.5 *μ*L reactions. The final reaction conditions were as follows: 1× PCR buffer, 1 mmol/L MgCl_2_, 200 *μ*mol/L of each dNTP, 10 pmol of each of the forward and reverse primer, 1 U *Taq* DNA polymerase, and 50 ng genomic DNA template. The PCR was carried out in the BOECO TC‐PRO Thermal Cycler. The conditions for PCR amplification were as follows: 5 min at 95°C denaturation, 30 cycles for 30 sec at 95°C, 30 sec at 50–60°C, and 30 sec at 72°C, followed by extension at 72°C for 40 min. PCR products were pooled and run against a Genescan™ 500 LIZ™ internal size standard on an ABI 3130 Genetic Analyzer (Applied Biosystems, Foster City, CA). Samples were genotyped using GeneMapper v.4.0 (Applied Biosystems, Inc.).

### SNP genotyping

A total of 31 SNP markers were typed as described in Labuschagne et al. (2012). These markers were developed via screening of a random genomic library. Thus far, these are the only SNP markers that have been reported for the species. Amplification was achieved using Dream Taq™ Green PCR Master Mix (2×) supplied by Thermo Scientific, Lithuania. The PCR mix for each locus contained 12.5 *μ*L of 2× Dream Taq™ PCR Master Mix (10× Dream Taq™ buffer, dATP, dCTP, dGTP, and dTTP, 0.4 mmol/L each, 4 mmol/L MgCl_2,_ and 1.25 U Dream Taq™ polymerase), 1 *μ*L [10 *μ*mol/L] of each primer, 50 ng of template DNA, and nuclease‐free water to reach a final volume of 25 *μ*L. Sequencing of resulting amplicons was conducted by Inqaba Biotechnical Industries (Pty) Ltd using the ABI Big Dye V3.1 kit and the ABI 3500XL Genetic Analyzer. Sequence data were screened and aligned using the Main workbench from CLC Bio (Denmark).

### Parentage analysis

Parentage assignment was evaluated with likelihood‐ and exclusion‐based approaches, using the MS and SNP data sets individually and combined. To assign parentage using a likelihood approach, we used the software program CERVUS v3.03 (Kalinowski et al. 2007). The program uses multilocus parental exclusion probabilities (Selvin 1980) and pairwise likelihood to assign parent pairs to offspring. CERVUS calculates the log‐likelihood of each candidate parent being the true parent relative to an arbitrary individual and then calculates the difference between the two most likely parents (Delta, Δ). Critical values of Δ are determined by computer simulation. Using the real data for allele frequencies, simulation parameters were set at 10,000 offspring, with 100% of candidate parents sampled and a total proportion of loci typed over all individuals of 0.99, mistyping error rates = 0.01 and likelihood calculation error rates = 0.01, permitting two unscored loci. Strict confidence was set to 95%, while the relaxed confidence level was 80%. CERVUS was also used to calculate the summary statistics including allele number at each locus (*k*), observed heterozygosity (*H*_obs_), expected heterozygosity (*H*_exp_), polymorphic information content (PIC), average nonexclusion probability for one candidate parent (NE‐1P), average nonexclusion probability for one candidate parent given the genotype of a known parent of the opposite sex (NE‐2P), and significance of deviation from Hardy–Weinberg equilibrium (HW). Parentage assignment using exclusion was performed in PARFEX v1.0 (Sekino and Kakehi 2012). The exclusion method examines incompatibilities between putative parents and offspring genotypes based on Mendelian principles. Parentage assignments were made for zero, one, and two mismatches. PARFEX was further used to calculate a minimum marker set required for optimal parentage using the given data set through the PFX_Mchoice macro. The known parental genotypes are used to simulate offspring genotypes, which are then subjected to exclusion‐based parentage testing with successive one‐by‐one addition of higher‐ranked markers from which the cumulative success rate of parentage allocation is obtained (Sekino and Kakehi 2012). Markers are ranked through one of three statistics (proportion of unique alleles, polymorphic information content [PIC], and exclusion probability) and the success rate of parentage allocation defined as the number of simulated offspring whose true parental pair is unambiguously identified divided by the total number of offspring (Sekino and Kakehi 2012).

## Results

**Figure 3:**
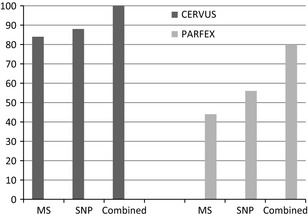
Percentage correct parent–offspring assignments for all data sets using CERVUS and PARFEX.

**Figure 4:**
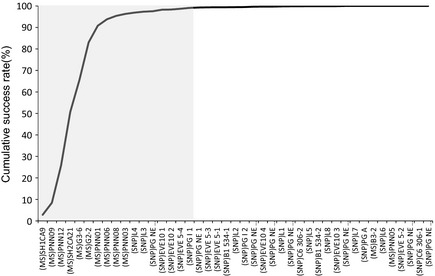
The cumulative success rate of parentage assignment based on exclusion with markers ranked on PIC value. The gray area encompasses all loci required to reach a 100% probability of assigning a correct parent–offspring relationship.

The 33 individuals used in this study represented 17 males and 16 females according to the studbook data. Molecular sexing using the CHD gene verified the gender of all individuals. All samples were successfully genotyped, with the exception of one MS marker for one sample, while the SNP data set had five SNPs missing, affecting three samples. Genotyping was conducted once on all samples and was not repeated in cases of no amplification. Lack of amplification may be due to low sample quality. In total, 62 alleles were found over all 12 MS loci, with a mean PIC of 0.54 (Table 1). Thirty‐one SNPs were identified with a mean PIC of 0.23 (Table 2). Deviations from HW and gametic disequilibrium were not observed for any of the markers. The NE‐1P (average nonexclusion probability for one candidate parent) for the SNP set was 0.2126, 0.0389 for the MS set and 0.0082 for the combined data set. The SNP marker set presented with a mean expected heterozygosity of 0.2803, whereas the MS marker set was 0.5952. For the 33 samples collected, 25 parent–offspring relationships can be made from the studbook data (Figs. 1, 2). Among these relationships, nine are sire/dam/offspring trios (Fig. 1C and F–J), seven single parent/offspring pairs (Fig. 1A–E), four sets have full‐siblings (Fig. 1A, B, H and J), and two family groups include previous generations (Fig. 1C and H). All potential maternal and paternal candidates were used in parentage analyses with no prior exclusions made with candidate subsets. Using the MS data set in PARFEX (Table 4), only 11 of the 25 relationships could be correctly assigned using the exclusion method (Fig. 3). The SNP data set performed better with 14 of the 25 relationships being assigned. When combining both data sets, 20 of the relationships could be assigned using the exclusion method (Figs. 1, 3). By applying the MS data in PARFEX, correct parents were mostly excluded due to a high number of mismatches, while in the SNP data set, there were often not enough differences to discern false parents from true parents (Fig. 2; Tables 3 and 4). Using the MS data set in CERVUS (Fig. 1; Table 3), 21 of the relationships could be correctly assigned when using a likelihood method. The SNP data set assigned 22 correct relationships with the same methodology (Fig. 1). When combining both data sets in CERVUS, all 25 relationships were correctly assigned (Fig. 1). Incorrect assignments with the MS data were limited to three family groups (Fig. 1B, D, and E), all single parent–offspring groups. All four assignments had low LOD scores (Fig. 1). Incorrect assignments with the SNP data were limited to two family groups (Fig. 1I and J). The incorrect assignment in group I was made with 95% confidence, while both assignments in group J had 80% confidence. In contrast with the CERVUS MS data, the correct parent was assigned to PNN156 in group B. Dam PNN149 was the closest match although it contained two mismatches (Table 4). The remaining incorrect CERVUS assignments were also incorrect in PARFEX. A similar disparity was noted in the SNP data set where both parents are correctly assigned in group J for offspring PNN96 using PARFEX (Fig. 2). The incorrect assignments for groups I and J in CERVUS were nonexcluded in PARFEX. Several parents could be assigned without mismatches (Table 4). PFX_Mchoice only reached 99% accumulative success rate when ranking markers through exclusion probability or proportion of unique alleles. Using exclusion probability, 99% accumulative success rate was reached with 15 markers (10 MS and five SNPs). Using only these 15 markers, 22 of the 25 relationships could be assigned correctly. By ranking markers through the proportion of unique alleles, 99% accumulative success was achieved with 22 markers (11 MS and 11 SNPs). Using the 22 marker subset, 23 of the 25 relationships could be assigned accurately. Ranking markers using PIC resulted in a 100% accumulative success rate with 34 markers (10 MS and 14 SNPs) (Fig. 4). All 25 relationships were assigned correctly when using these markers.

**Table 1:** Parameters of genetic information content of 12 microsatellite loci estimated from ex situ population of African penguin. *k* = number of alleles; *N* = number of samples; *H*_obs_ = observed heterozygosity; *H*_exp_ = expected heterozygosity; PIC = polymorphic information content; NE‐1P = average nonexclusion probability for one candidate parent; and NE‐2P = average nonexclusion probability for one candidate parent given the genotype of a known parent of the opposite sex

Locus	k	N	*H*_obs_	*H*_exp_	PIC	NE‐1P	NE‐2P
G2‐2	5	33	0.697	0.695	0.627	0.740	0.577
SH1CA9	10	33	0.788	0.779	0.746	0.593	0.409
SH2CA21	7	33	0.667	0.740	0.688	0.672	0.495
B3‐2	3	33	0.152	0.172	0.161	0.986	0.915
G3‐6	7	33	0.636	0.730	0.669	0.697	0.526
PNN01	4	33	0.727	0.675	0.595	0.773	0.621
PNN03	5	33	0.394	0.424	0.383	0.909	0.773
PNN06	4	33	0.636	0.656	0.578	0.786	0.634
PNN08	4	33	0.697	0.656	0.584	0.781	0.624
PNN09	6	33	0.758	0.769	0.717	0.645	0.468
PNN12	5	32	0.875	0.730	0.671	0.695	0.523
PNN05	2	33	0.121	0.116	0.107	0.994	0.946
Mean	5.17			0.5952	0.5439	0.0389	0.0024

**Table 2:** Parameters of genetic information content of 31 single nucleotide polymorphisms estimated from ex situ population of African penguin. *k* = number of alleles; *N* = number of samples; *H*_obs_ = observed heterozygosity; *H*_exp_ = expected heterozygosity; PIC = polymorphic information content; NE‐1P = average nonexclusion probability for one candidate parent; and NE‐2P = average nonexclusion probability for one candidate parent given the genotype of a known parent of the opposite sex

Locus	SNP	k	N	*H*_obs_	*H*_exp_	PIC	NE‐1P	NE‐2P
PG NE 15	P110 NE‐15‐1	2	33	0.061	0.060	0.057	0.998	0.971
	P110 NE‐15‐2	2	33	0.303	0.339	0.278	0.944	0.861
PG NE 12	P110 NE‐12‐1	2	31	0.032	0.032	0.031	0.999	0.984
	P110 NE‐12‐2	2	31	0.129	0.228	0.200	0.975	0.900
PG NE 11	P110 NE‐11‐1	2	33	0.333	0.416	0.326	0.916	0.837
	P110 NE‐11‐2	2	33	0.394	0.357	0.290	0.938	0.855
	P110 NE‐11‐3	2	33	0.273	0.239	0.208	0.972	0.896
PG NE 1	P110 NE 1	2	33	0.485	0.451	0.346	0.901	0.827
PG EVE 5	P110 EVE 5‐1	2	33	0.333	0.416	0.326	0.916	0.837
	P110 EVE 5‐2	2	33	0.061	0.060	0.057	0.998	0.971
	P110 EVE 5‐3	2	33	0.485	0.429	0.333	0.911	0.833
	P110 EVE 5‐4	2	33	0.515	0.441	0.340	0.906	0.830
C6 306	P110 C6‐306‐1	2	33	0.030	0.030	0.029	1.000	0.985
	P110 C6‐306‐2	2	33	0.273	0.282	0.239	0.961	0.880
B1 534	P110 B1‐534‐1	2	33	0.424	0.403	0.318	0.921	0.841
	P110 B1‐534‐2	2	33	0.303	0.261	0.224	0.967	0.888
PG L	P110‐L‐1	2	33	0.273	0.239	0.208	0.972	0.896
	P110‐L‐2	2	33	0.242	0.373	0.300	0.933	0.850
	P110‐L‐3	2	33	0.515	0.478	0.360	0.889	0.820
	P110‐L‐4	2	33	0.576	0.506	0.374	0.876	0.813
	P110‐L‐5	2	33	0.152	0.142	0.130	0.990	0.935
	P110‐L‐6	2	33	0.091	0.088	0.083	0.996	0.958
	P110‐L‐7	2	33	0.121	0.168	0.152	0.986	0.924
	P110‐L‐8	2	33	0.242	0.216	0.190	0.977	0.905
PG I	P110 I‐1	2	33	0.424	0.373	0.300	0.933	0.850
	P110 I‐2	2	33	0.364	0.302	0.253	0.956	0.873
PG A	P110‐A1	2	32	0.094	0.091	0.085	0.996	0.957
PG EVE 10	P110 EVE10‐1	2	33	0.455	0.416	0.326	0.916	0.837
	P110 EVE10‐2	2	33	0.394	0.388	0.309	0.927	0.845
	P110 EVE10‐3	2	33	0.152	0.142	0.130	0.990	0.935
	P110 EVE10‐4	2	33	0.333	0.321	0.266	0.950	0.867
Mean					0.2803	0.2280	0.2126	0.022

**Table 3:** CERVUS parentage assignments. Brackets indicate correct assignment; * = 95% confidence; + = 80% confidence; incorrect assignments marked in gray

Offspring	Combined data sets				Microsatellites				SNPs			
	Candidate mother	Pair LOD score	Candidate father	Pair LOD score	Candidate mother	Pair LOD score	Candidate father	Pair LOD score	Candidate mother	Pair LOD score	Candidate father	Pair LOD score
PNN147	(PNN168)*	0.65	n/a	n/a	(PNN168)	−2.81	n/a	n/a	(PNN168)*	3.46	n/a	n/a
PNN156	(PNN149)*	4.18	n/a	n/a	PNN135	−3.94	n/a	n/a	(PNN149)*	4.60	n/a	n/a
PNN165	(PNN141)*	5.57	n/a	n/a	(PNN141)*	3.33	n/a	n/a	(PNN141)*	2.24	n/a	n/a
PNN161	(PNN149)	−2.36	n/a	n/a	PNN168	−7.12	n/a	n/a	(PNN149)	1.10	n/a	n/a
PNN175	(PNN141)*	8.49	n/a	n/a	(PNN141)*	5.79	n/a	n/a	(PNN141)*	2.70	n/a	n/a
PNN113	n/a	n/a	(PNN69)*	0.85	n/a	n/a	PNN80	−2.08	n/a	n/a	(PNN69)*	2.30
PNN122	n/a	n/a	(PNN74)	−2.48	n/a	n/a	PNN80	−3.84	n/a	n/a	(PNN74)*	4.46
PNN37	(PNN43)*	9.44	(PNN39)*	8.60	(PNN43)*	4.97	(PNN39)*	7.76	(PNN43)*	4.48	(PNN39)+	0.84
PNN40	(PNN35)*	3.68	(PNN41)*	8.34	(PNN35)	−1.55	(PNN41)*	2.97	(PNN35)*	5.24	(PNN41)*	5.37
PNN42	(PNN44)*	0.60	(PNN45)*	2.21	(PNN44)*	5.04	(PNN45)*	2.34	PNN168*	1.73	(PNN45)	−0.13
PNN75	(PNN81)*	4.19	(PNN80)	1.18	(PNN81)*	1.34	(PNN80)	−2.13	(PNN81)*	2.86	(PNN80)*	3.31
PNN80	(PNN82)*	4.70	(PNN83)*	0.48	(PNN82)*	1.45	(PNN83)	−1.32	(PNN82)*	3.25	(PNN83)*	1.80
PNN47	(PNN70)*	8.72	(PNN68)*	3.30	(PNN70)*	5.59	(PNN68)*	3.26	(PNN70)*	3.13	PNN69+	0.94
PNN76	(PNN81)*	6.69	(PNN80)*	7.31	(PNN81)*	4.11	(PNN80)*	1.18	(PNN81)	2.58	(PNN80)*	6.13
PNN96	(PNN70)*	0.78	(PNN68)	−6.39	(PNN70)*	0.39	(PNN68)	−6.60	PNN44+	1.42	(PNN68)+	0.21
PNN178	(PNN135)	−1.14	(PNN147)*	9.42	(PNN135)	−1.85	(PNN147)	−1.15	(PNN135)+	0.72	(PNN147)*	10.57

**Table 4:** Exclusion‐based (PARFEX) parentage assignments. Brackets indicate true parent

Offspring	Mismatches	Combined data sets		Microsatellites		SNPs	
		Candidate mother	Candidate father	Candidate mother	Candidate father	Candidate mother	Candidate father
PNN147	0		n/a		n/a	(PNN168)	n/a
	1		n/a		n/a		n/a
	2	(PNN168)	n/a	PNN35, PNN135	n/a	PNN43	n/a
PNN156	0		n/a		n/a	PNN81, PNN135, (PNN149)	n/a
	1	(PNN149)	n/a		n/a	PNN44, PNN141,	n/a
	2	PNN135	n/a	(PNN149)	n/a		n/a
PNN165	0	(PNN141)	n/a	(PNN141)	n/a	PNN35, PNN44, PNN81, PNN135, (PNN141), PNN168	n/a
	1		n/a		n/a		n/a
	2	PNN135	n/a		n/a		n/a
PNN161	0		n/a		n/a	(PNN149)	n/a
	1		n/a		n/a	PNN35, PNN82	n/a
	2	(PNN149)	n/a		n/a	PNN43, PNN81, PNN135, PNN168	n/a
PNN175	0		n/a		n/a	(PNN141)	n/a
	1		n/a		n/a		n/a
	2	(PNN141)	n/a	PNN43, PNN135, (PNN141)	n/a		n/a
PNN113	0	n/a		n/a		n/a	PNN68, (PNN69)
	1	n/a		n/a		n/a	
	2	n/a		n/a	PNN45, PNN80	n/a	PNN39, PNN41, PNN45
PNN122	0	n/a	PNN45	n/a	PNN45	n/a	PNN39, PNN45, (PNN74)
	1	n/a	PNN39, (PNN74)	n/a	PNN39	n/a	
	2	n/a		n/a	PNN80	n/a	
PNN37	0					(PNN43)	(PNN39)
	1					PNN44, PNN168	
	2	(PNN43)	(PNN39)	(PNN43)	(PNN39)	PNN70, PNN81	PNN68, PNN69
PNN40	0					(PNN35), PNN82	(PNN41), PNN45
	1					PNN44, PNN168	PNN68
	2	(PNN35)	(PNN41)				PNN39, PNN74
PNN42	0	(PNN44),	(PNN45)	(PNN44)	(PNN45)	PNN43, (PNN44), PNN168	(PNN45)
	1					PNN81, PNN135	PNN68
	2	PNN81;	PNN41	PNN81, PNN82	PNN68	PNN70, PNN82	PNN69, PNN83, PNN147
PNN75	0		PNN83		PNN83	(PNN81), PNN149	(PNN80), PNN83
	1	(PNN81); PNN68	(PNN80)		PNN147, (PNN80)	PNN82, PNN135	PNN45, PNN68
	2			PNN43		PNN44	PNN39, PNN41, PNN45, PNN74
PNN80	0				PNN68	(PNN82)	
	1	(PNN82)		(PNN82)		PNN81, PNN168	(PNN83)
	2		(PNN83), PNN68		PNN74	PNN43, PNN44, PNN135	PNN68
PNN47	0	(PNN70)	(PNN68)	(PNN70)	(PNN68)	(PNN70), PNN168	PNN45, (PNN68), PNN69
	1	PNN81	PNN69			PNN43, PNN44	
	2	PNN43, PNN135, PNN168	PNN45	PNN82, PNN168	PNN39, PNN45, PNN74, PNN83	PNN81, PNN82, PNN135, PNN141	PNN39, PNN41
PNN76	0				PNN83	(PNN81)	(PNN80)
	1	(PNN81)	(PNN80)	(PNN81)	(PNN80)	PNN82, PNN135, PNN149	PNN68
	2	PNN82	PNN68			PNN44	PNN41, PNN45, PNN69, PNN83
PNN96	0					(PNN70)	(PNN68)
	1	(PNN70)		(PNN70)		PNN44, PNN81, PNN168	PNN45, PNN74
	2		(PNN68)		(PNN68), PNN69, PNN74	PNN82, PNN135, PNN141, PNN149	PNN69
PNN178	0					(PNN135)	(PNN147)
	1	(PNN135)	(PNN147),		PNN45;PNN68	PNN34, PNN44, PNN81, PNN141, PNN149, PNN168	PNN41, PNN45, PNN68, PNN69
	2		PNN68	(PNN135); PNN141	(PNN147); PNN83		

## Discussion

As inaccuracies in the studbook can have implications on future genetic and demographic analysis and management of the captive population, a suitable validated marker set for genetic parentage verification is an important tool for captive management (Ivy and Lacy 2010). Such a marker set may not only exclude incorrectly recorded parents, but also help in assigning the correct individuals if sampled. We have described and verified a set of genetic markers for ascertaining parentage and sibling relationships in African penguins. Few published studies have investigated parentage or paternity in penguins, and to our knowledge, none have used SNP markers. Seven MS markers (including one, B3‐2, employed in the present study) yielded a general exclusion probability (mother known) of 0.99 for little penguins (Billing et al. 2007), and eight MS markers (including one used in the present study – Sh1Ca9) yielded paternity exclusions of 0.94–0.99 for captive Adelie penguins (Sakaoka et al. 2014).

Concerning the discrimination power of both types of markers, MS and SNP, as expected, the MS markers with multiple alleles possible at each locus had an overall higher PIC value. Both marker sets had 62 independent alleles. However, with more loci, the optimized SNP marker set performed better than the MS marker set using both the exclusion and likelihood parental assignment methods. This study has indicated that the number of loci and their heterozygosity level may influence the power of markers for parentage exclusion approaches more than the number of independent alleles (Morin et al. 2004; Hauser et al. 2011). The power of molecular markers is also influenced by genotyping error (Kalinowski et al. 2007). The generally low error rate for SNPs is a definite advantage for parentage over the higher rates reported for MS markers (Walling et al. 2010; Hauser et al. 2011). However, as each locus adds linearly to the multilocus error, but provides diminishing information for parentage, even low error rates may become problematic as the number of loci screened becomes very large (Christie 2010; Hauser et al. 2011). The optimum number of loci should therefore be determined in preliminary experiments where the number of SNPs required may be less than commonly assumed (Christie 2010; Hauser et al. 2011). In the current study, we used PFX_Mchoice to establish whether a smaller subset of markers would achieve the same assignment power over the full combined marker set. A subset of 34 markers consisting of 10 MS markers and 14 SNP markers were identified that could accurately allocate all 25 parent–offspring relationships identified. Such a priori knowledge about a minimum set of markers providing a high resolution of parentage assignment helps reduce the experimental cost and labor involved in the subsequent parentage testing.

As parentage inference is not concerned with inference of evolutionary history, ascertainment bias through discovery, in particular populations or genomic regions, does not bias the results of parentage inference (Anderson and Garza 2006). In effect, such ascertainment typically leads to an overrepresentation of SNPs at intermediate allele frequencies, an advantage in parentage inference (Anderson and Garza 2006). Those SNP markers with minor allele frequencies of 0.5 provide the most power for parentage inference, although little additional power is gained above frequencies of 0.4 (Anderson and Garza 2006). Choosing SNP markers with allele frequencies above 0.2 can achieve higher assignment power with fewer loci. Among the current 34 SNP markers, only 16 have heterozygosity above 0.3. Replacing the markers falling below these ranges with new marker with higher ranges may greatly improve the number of loci versus assignment power ration as well as provide a SNP‐only marker set that takes full advantage of SNP marker benefits over MS markers. Advantages including low error rates, ease of typing, low‐cost high‐throughput genotyping, and SNP genotypes that are easily standardized across laboratories are all important factors for a multi‐institutional studbook.

## Conclusion

The aim of this study was to generate molecular genetic information to verify/complement studbook‐based pedigree data from ex situ populations of African penguins. In addition, we compared the relative and combined utility of MS and SNP markers for parentage assignment. We found that a combined subset of these two types of markers attained a >99% correct cumulative parentage assignment probability. Information derived from this “optimal” marker set will be useful for future captive management of African penguins.

## Conflict of Interest

None declared.
